# A Systematic Review of the Health Impacts of Mass Earth Movements (Landslides)

**DOI:** 10.1371/currents.dis.1d49e84c8bbe678b0e70cf7fc35d0b77

**Published:** 2015-04-30

**Authors:** Iain T R Kennedy, Dave N. Petley, Richard Williams, Virginia Murray

**Affiliations:** Extreme Events and Health Protection Section, Centre for Radiation Chemical and Environmental Hazards, Public Health England, UK; School of Environmental Sciences, University of East Anglia, Norwich, UK; Welsh Institute for Health and Social Care, University of South Wales, Pontypridd, UK; Extreme Events and Health Protection, Public Health England, London, UK

## Abstract

**Background. **Mass ground movements (commonly referred to as ‘landslides’) are common natural hazards that can have significant economic, social and health impacts. They occur as single events, or as clusters, and are often part of ‘disaster’ chains, occurring secondary to, or acting as the precursor of other disaster events. Whilst there is a large body of literature on the engineering and geological aspects of landslides, the mortality and morbidity caused by landslides is less well documented. As far as we are aware, this is the first systematic review to examine the health impacts of landslides.

**Methods.** The MEDLINE, EMBASE, CINAHL, SCOPUS databases and the Cochrane library were systematically searched to identify articles which considered the health impacts of landslides. Case studies, case series, primary research and systematic reviews were included. News reports, editorials and non-systematic reviews were excluded. Only articles in English were considered. The references of retrieved papers were searched to identify additional articles.

**Findings.** 913 abstracts were reviewed and 143 full text articles selected for review. A total of 27 papers reporting research studies were included in the review (25 from initial search, 1 from review of references and 1 from personal correspondence). We found a limited number of studies on the physical health consequences of landslides. Only one study provided detail of the causes of mortality and morbidity in relation a landslide event. Landslides cause significant mental health impacts, in particular the prevalence of PTSD may be higher after landslides than other types of disaster, though these studies tend to be older with only 3 papers published in the last 5 years, with 2 being published 20 years ago, and diagnostic criteria have changed since they were produced.

**Discussion.** We were disappointed at the small number of relevant studies, and the generally poor documentation of the health impacts of landslides. Mental health impacts were better documented, though some of the studies are now quite old. Further research on the health impacts of landslides needs to be undertaken to support those responding to landslide disasters and to aid disaster risk mitigation advocacy.

## Introduction

In a progressively more populated world, landslides are increasing in frequency; a trend that is expected to continue over the remainder of the 21^st^ Century^1^. Mass movements of geological materials (which in this paper we will, in common with usual preferred practice in the natural science domain, term ‘landslides’) are one type of the most common of all natural hazards. Instances have been recorded in every global environment in which slopes are present. They occur singly or in clusters in both space and time, especially when they are triggered by other natural hazards, such as tropical cyclones and earthquakes. Human bodies are poorly equipped to withstand the high-energy nature of the mechanisms involved in landslides such that a large proportion of affected people are killed. However, until now, there has been no systematic review of the health impacts associated with mass movements of geological materials (i.e of landslides).

A landslide may be defined as a “*downhill and outward movement of slope-forming materials under the influence of gravity”^2^*. Typically, landslides are triggered by external processes such as rainstorms, earthquakes, snowmelt and slope disturbance by humans. They include a range of phenomena, including rockfalls, debris flows and rock avalanches, but exclude avalanches that are composed primarily of ice and/or snow, and flows that are composed predominantly of water. Landslides are commonly categorised in terms of movement mechanism and dominant material type (Figure 1).

**Classification of landslides by material and mechanism:**
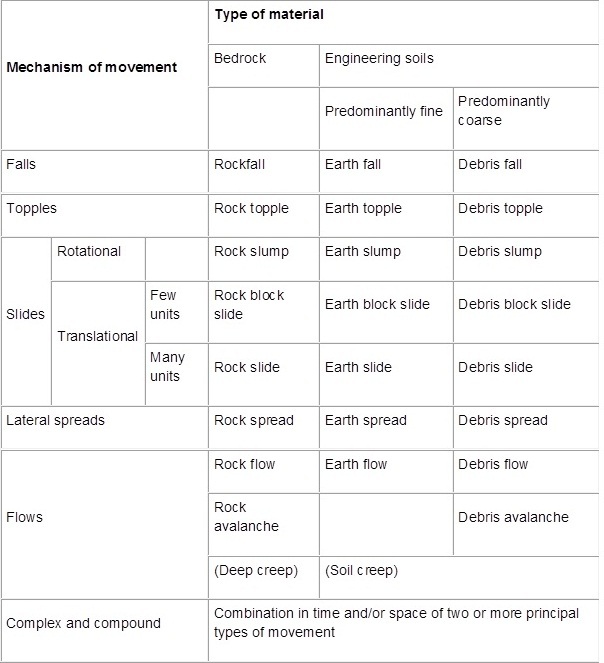
From Special Report 176: Landslides: Analysis and Control. Varnes, D., Chapter 2: Slope Movement Types and Processes, Figure 2.1. Copyright, National Academy of Sciences, Washington, D.C., 1978. Reproduced with permission of the Transportation Research Board.

There are many problems associated with the compilation of mortality data from landslides, including, but not limited to^3^:

- The location of many landslides in remote, often highly mountainous, areas in poor countries in which levels of reporting of lower impact events is likely to be variable;
- Misidentification of landslides, for example considering a debris flow to be a flood, leading to them being incorrectly recorded;
- The co-occurrence of landslides with another (triggering) process, such as an earthquake, leading to the cause of death being incorrectly recorded;
- The difficulty of tracking down the occurrence of post-event mortality.

Consistently, until recently, the number of fatalities from landslides worldwide have been incompletely estimated^4^. Petley compiled a dataset of landslide fatalities for the period 2004-2010, which included 80,058 deaths (estimated error (one standard deviation) is 5% lower to 20% higher than the point estimate) from mass earth movements. He reported that 47,736 of those deaths were attributable to landslides triggered by earthquakes^4^. In comparison and according to the UN disaster database EM-DAT (2012), only 2,923 people were recorded as being killed in wet and dry mass movements in the same period. Previously, Guha-Sapir has explored some of the reasons for the difficulties in recording the impacts of natural disasters and the variability between disaster databases^5^.

The majority of human losses from landslides occur in mountainous, less developed countries, with particular hotspots along the southern edge of the Himalayan Arc (i.e. in the mountainous areas of northern India, northern Pakistan, Nepal, Bhutan and western Bangladesh), Central America, the Caribbean, Colombia, the Philippines and Indonesia^4^.

In addition, landslides have a multiplying effect on losses from other disasters. Thus, for example, in both the 2005 Pakistan and 2008 China earthquakes rescue and recovery operations were hindered greatly by landslides that blocked key access routes, most notably roads, which meant that survivors of the earthquakes succumbed to their injuries before they could receive effective medical care^6^.

Landslides also generate very high levels of economic loss, although, at present, these losses are poorly quantified. Schuster and Highland (2001) estimated losses of up to US$1 billion per annum in Canada and $2 billion in the USA^7^. The economic costs of landslides may also follow a different pattern to that of mortality, because the highest monetary losses are likely to occur in upland areas of developed countries that have significant financial assets.

Despite these impacts, there has been no previous systematic review of the health impacts of landslides. Considerable engineering and geological work has been done to understand the spatial and temporal distributions of landslides, and the physical mechanisms through which they occur, but almost no equivalent work has been undertaken to synthesise information from the perspective of people’s health needs. We believe that a better understanding of the health implications of landslides will provide information, which, if properly used, could reduce the risks caused by disasters and enhance rescues of, and treatment for casualties from landslides.

## Methods

A study protocol is available from the authors. We searched MEDLINE, EMBASE, CINAHL, the Cochrane library and SCOPUS databases, where possible using both free text and keyword searching. We used the following search string:

Landslide* OR landslip* OR rockfall* OR earthflow* OR mudslide* OR mudflow* OR debris flow* OR rockslide* OR rock avalanche* OR earth flow* OR debris slide* OR sturzstrom*

AND

Mortality OR morbidity OR casualt* OR death* OR injur* OR epidemiol* OR accident* OR burden*

We searched databases from the first available date (MEDLINE: 1950, EMBASE: 1980; CINAHL: 1981) to the beginning of November 2012. Due to the large number of papers on geological rather than specific health impacts, our search on the SCOPUS database was limited to papers published since 1995. The full search strategy for one database is shown in Figure 2

**Detail of search strategy as performed in EMBASE:**
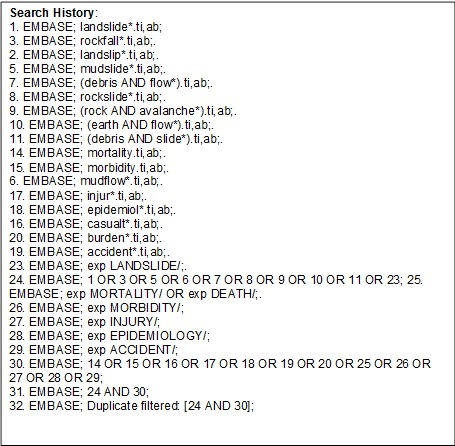

The inclusion criteria were:

- Studies that quantify or estimate the health impacts of mass ground movements (to include landslides, mudslides and rockfalls) such as mortality, morbidity, mental health;
- Review articles, primary research including epidemiological studies, case series and case reports.

The exclusion criteria were:

- Studies on mass movements of snow and ice (avalanches), flooding and submarine landslides;
- Studies related to technical or geological matters;
- Studies that solely simply state a fatality figure without further description of, for example, cause of death, mechanism of injury, or otherwise quantifying health and wellbeing or which further analyse the data by examining trends, risk factors, etc.;
- Editorials and news articles;
- Studies in languages other than English.

The titles and, where available, the abstracts found by the searches were independently checked by at least two authors of this review. We attempted to retrieve the full text of all articles that were identified as potentially eligible by at least one author. We screened the full text articles to ascertain as to whether they met the inclusion or exclusion criteria. Data was extracted from the eligible studies, compiled and tabulated.

We checked the reference lists in each eligible paper to identify any relevant papers not retrieved from initial search.

We have included a copy of the PRISMA checklist in Appendix 1.

## Results

A flow chart describing the results of the literature search is shown in Figure 3

**Flowchart of search strategy:**
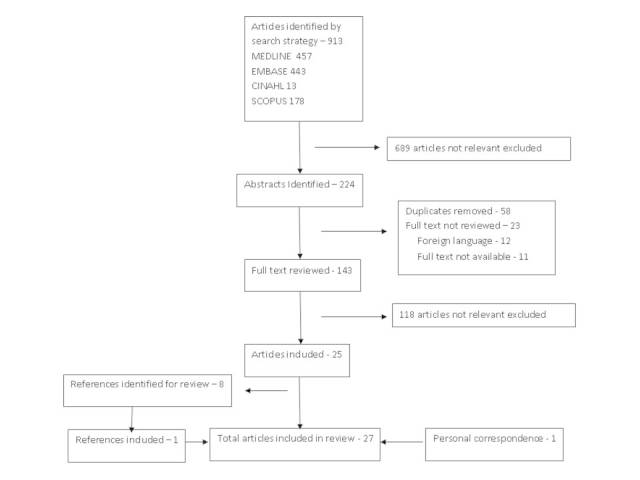

We divide our findings from this search and review of the papers that it produced into the following sections: general findings, estimates of mortality, causes of mortality and morbidity, crush syndrome, post disaster infectious disease, the psychosocial effects and impacts on people’s mental health and impacts on healthcare.

**General findings**

We identified few peer-reviewed publications on health impacts from landslides. Only a small proportion of the extensive literature on landslides related to health, with most studies focusing on geological and technical aspects. Figure 4 contains details of the 27 papers that met our inclusion criteria. In general, the papers report studies that are limited to: specific catastrophic events that involved major loss of life or destruction of infrastructure; events that received media attention; and a small number of case studies of events with lower levels of impact. The largest group of papers relates to impacts on people’s psychosocial experiences and needs, and their mental health. Studies on mortality have concentrated primarily on mortality rates. Only a few papers report studies that mention specific mechanisms of mortality and morbidity.

**Papers included in review:**
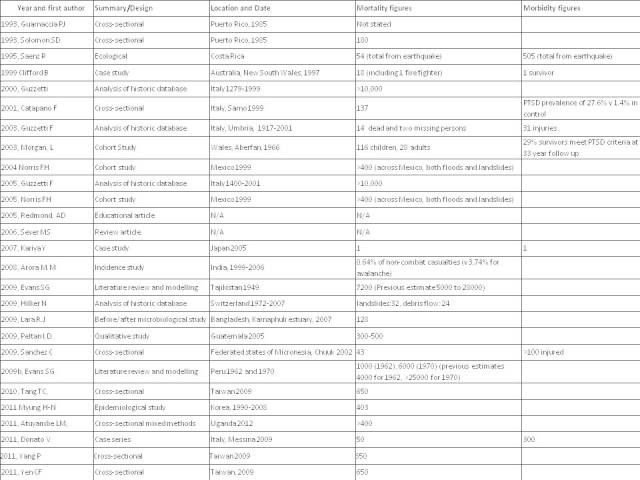

The papers we reviewed emphasise the very significant, short-term health impacts of landslides. Thus, Evans *et al. *document two landslides at Huascaran in Peru^8^; one in 1962 and one in 1970, that, together, claimed about 7,000 lives. The large number of deaths may be attributed to the volume of mobile material (58 million m^3^in the 1970 event), the high rates of movement (the peak velocity is thought to have been about 80 m/sec in 1970), the long run out distance (the resulting debris flow travelled 180 km until it reached the Pacific Ocean), and the vulnerability of the population. In both cases, the landslides are thought to have killed about 90% of the population of the towns that they struck.

Despite these levels of human mortality, data on the impacts of landslides tend to focus on economic loss rather than social costs. Thus, for example, one study of the impacts of landslides in an alpine country, Switzerland, over a 35-year period calculated that landslides contribute 11% of the total financial cost of disasters in the country. However, the study demonstrated that there is little correlation between the financial costs and the health impacts of landslides^9^.

**Estimates of mortality**

Until Petley’s study^4^, many datasets grossly under-represented total loss of life in landslides. However, in some cases, the effects of individual events has also been over-estimated in the literature, especially for mass mortality events. Evans *et al* 8 ^,^ 10 re-evaluated landslides in Tajikistan in 1949, and Peru in 1962 and 1970, that caused mass mortalities. They estimated the fatality count to be around 7,200 after the Tajikistan event, whereas previous estimates ranged as high as 28,000 deaths, whilst their research on the two events in Peru suggests accurate estimates to be 1,000 fatalities in 1962 (previous estimate 6,000 deaths) and 6,000 deaths in 1970 (previous estimates of more than 25,000 fatalities). The likely cause of these estimates is an over-reliance on contemporaneous reporting and failure to consider available evidence regarding population densities in the affected areas.

Italy, which is the country in Europe that is most affected by landslides, is rare in having a comprehensive nationwide database for landslides that has been analyzed extensively by Guzzetti *et al* 11 ^,^ 12 ^,^ 13 . In 2000, they showed that the Italian average of 59.4 deaths or missing people per year induced by landslides is higher than after similar events in Australia, Canada, Norway Portugal, Spain, UK and the USA. These rates are lower than the averages for China, Japan, Peru and the Southern Andes^11^. This database has also been used to analyse the risk of mortality associated with landslides in Italy, which indicated an average of 7.33 events causing fatalities per year for the period 1900 to 2000. The average landslide mortality rate was 0.084 deaths per 100,000 population, based on the enumerated census population, for the period 1861 to 2001 (the period for which census data is available to allow the calculation). This is compared to an overall mortality rate from all disease of 967.4 deaths per 100,000 population. However these calculations are very sensitive to changes in population size and geographical distribution, so they should be used with considerable caution^13^.

A further country-specific study covering deaths from meteorological causes in Korea from 1990 to 2008^14^, demonstrated that 19.7% of deaths (403 out of 2,045) during meteorological disasters were due to landslides. Of these, 251 deaths were from landslides caused by flooding (representing 26% of all flooding deaths) and 149 were from landslides following typhoons (19.9% of typhoon deaths). These differing percentages demonstrate the importance of precision in defining and recording the cause of death when considering disaster-related fatalities.

In a different setting, a study by the Indian Army^15^ demonstrated that 0.64% of non-combat deaths occurred as a result of landslides, which compares to 3.74% of casualties who were killed by avalanches. These findings reflect the high levels of hazard that soldiers face in the disputed Siachen Glacier area on the border with Pakistan.

**Causes of mortality and morbidity**

Only one paper that describes a study of the causes of mortality and morbidity in landslides met our search criteria. This was a retrospective study of a landslide event, which included a review of death certificates, a cross-sectional survey, and interviews with survivors, and surrogates representing deceased persons^16^. The methodology of that study, including the use of surrogates, has potential to introduce bias into the results, though we note that surrogates have been used successfully in other research settings

This study examines the risk factors for mortality during landslides in Chuuk, Micronesia in 2002^16^. The causes of death for the 43 fatalities were found to be suffocation caused by being buried in the landslide (39/43), blunt trauma causing sudden death from multiple head injuries (1/43), delayed death from head injury (1/43), delayed death caused by sepsis from skull fracture (1/43), and delayed death caused by abdominal trauma and fractured pelvis (1/43). Generally, the injuries sustained by survivors were relatively minor; in total, 48 people required emergency room attendance, of whom 43 were admitted to hospital. The most common injuries were lacerations and contusions, and concussions and fractures were less common.

Possible risk factors for mortality were also analysed in the study. The analysis showed that being under the age of 15 was a statistically significant risk factor for increased mortality. Female gender and being inside a building when the landslide occurred were also factors that increased mortality, though this increase was not statistically significant. People being aware that landslides had recently occurred in the vicinity and their noticing natural warning signs lowered the risk of mortality. However, this study found no association between the size of the landslide or the slope angle and its impact on people’s health^16^.

Other studies have reported cases of injury associated with being struck by rock or other debris when the injured people are not buried by the mass movement^17^, while Guzzetti *et al.* 12 showed that, of 23 recorded fatalities or missing persons from landslides in the Umbria region of Italy, eight deaths were associated with failures in excavations or mines. They also reported that six of the remaining fifteen fatalities, occurred in a single event when a landslide struck a train. This finding highlights the additional risks that are associated with landslides affecting road and rail corridors^12 ^.

^12^There is very little information available about the relationship between the occurrence of fatalities and landslide type. Guzzetti et al^13^, found that 80% of deaths caused by landslides in Italy resulted from rapid events, such as debris flows and mudflows. The occurrence of fatalities in slower moving events (such as rotational landslides) was much lower. However, when slower moving landslides do cause fatalities, the number of lives lost in each event tends to be higher than for the more rapid events. Probably, this is because, frequently, they induce unanticipated collapses of buildings. The percentage of fatalities and injuries caused by each type of mass movement was calculated from the Italian landslide database. Rockfalls were found to account for 35% of injuries recorded in the database, but only 5% of fatalities, whereas rockslides and rock avalanches account for >20% of fatalities but 5% or less of the injuries^13^.

**Crush injuries**

Incorporation into a landslide can subject people’s bodies to significant levels of external loading from debris. Therefore, survivors are at risk from crush injury or crush syndrome. Crush injury can result in skin necrosis and bony injury, while crush syndrome is characterised by rhabdomyolysis, renal failure and hyperkalaemia^18^.

Crush syndrome is the result of slow and long compression of skeletal muscle, which can produce severe ischaemia and reperfusion injury. These injuries can result in kidney and other organ damage, consequent on the rhabdomyolysis, with development of multi-organ pathologies leading to death in severe cases. Sever *et al* 19 suggested that whilst crush syndrome is the second most common cause of death after natural disasters, it is treatable, particularly if caught early. Fluid resuscitation and dialysis are the mainstays of treatment^19^.

Donato *et al*. reported on the treatment of two patients recovered from a landslide. This study indicated that biomarkers can be used to identify the need for dialysis at an earlier stage, which could, in turn, be instrumental in improving survival following injury caused by crushing. The research is at an early stage^20^. However, with a short case-series being the focus of this paper, further research into the use of these biomarkers would be valuable.

**Infectious diseases after disasters**

Landslides have been associated with outbreaks of infectious diseases. A huge increase in the incidence of malaria was recorded after the earthquake and floods in Costa Rica in 1991. Depending on region, the peak in monthly reported rates was between 1,600% and 4,700% higher than the pre-earthquake rates. In part, the increased incidence of malaria was probably the result of an increase in mosquitoes caused by deforestation and changes in river flow patterns. These were, in turn, consequent on the landslides triggered by the earthquake^21^.

Atuyambe et al. have described their study at a camp for displaced peoples following a landslide in Eastern Uganda in which they used mixed quantitative and qualitative methods to assess water, sanitation and hygiene conditions^22^. The study found that that there was insufficient access to clean water or latrines, which was exacerbated by traditional beliefs and attitudes towards water and hygiene, with many residents returning to a river water source, despite it being contaminated and other water supplies being available. There was also a significant burden of infectious disease, although, given the hygiene concerns, there was, arguably, less diarrhoeal disease than might have been expected, with only 8.8% of respondents reporting a member of their household having those symptoms. However malaria (reported in 47.7% of respondents) and respiratory infections (reported in 58.3% of respondents) were far more common^22^.

Often, debris from mass movements enters water courses, which increases turbidity and changes other parameters. If landslides go through inhabited areas, they can also disrupt normal waste management and further pollute water supplies. This was seen in the Karnaphuli Estuary in Bangladesh after a landslide in May 2007 23. After the landslide, the combination of changes in turbidity and salinity, and the rise of waste levels in already polluted waters, resulted in an increase in bacterial growth, including a ten-fold increase in faecal coliforms. There was also an increase in *Vibrio cholerae* populations, though it was not as great as the increase following the cyclone one month earlier^23^. Note however that this was a microbiological study, and the effect of these increases on human health was not quantified.

**The Psychosocial and Mental Health Impacts**

The nine papers relating to the psychosocial and mental health impacts of landslides that were discovered through the initial search process, fall into three main categories. Two review psychosocial matters including psychosocial support and the moderating effects of family roles^24^^,^ 25. Six papers report studies of the prevalence of psychiatric disorders in survivors^26^^,^ 27 ^,^ 28 ^,^ 29 ^,^ 30 ^,^ 31 and a single paper describes responses to the needs of firefighters who intervened after a landslide^32^. Many of these papers also refer to impacts of landslides on people’s relationships and the effect this has on psychosocial and mental health. Children or adolescents were the subjects in three of the papers, though the studies concern different aspects of the same small cohort. A tenth paper, identified through communication with one of the authors, details long term follow-up of the survivors of the Aberfan disaster, one of the most significant landslide events in the UK^33^.

Catapano et al. report a controlled prevalence study among the survivors of the disaster in Sarno, Italy, in 1998. It was performed one year after the event and examined the occurrence of mental disorders in the aftermath of landslides using standardized self-completed questionnaires. Survivors were more than twenty times more likely than members of a control group to suffer from post-traumatic stress disorder (PTSD), with 27.6% of survivors meeting the diagnostic criteria for PTSD compared to 1.4% in the control group. One year after the disaster, PTSD symptoms were nearly universal in the population of Sarno, with 90% of the study sample having ‘cluster B’ symptoms, which are those PTSD symptoms relating to intrusive experiences. Scores of greater than 5 on the GHQ-30, a validated and well-used questionnaire, indicate a ‘probable’ mental disorder, were identified in 59% of subjects from Sarno compared to 35% in the control groups^26^. Whilst these figures are high compared to other disasters at this time point, the authors suggest that this is because the participants living in the ‘red area’ around Sarno remain exposed the risk of similar disasters, and have experienced multiple moderate mudslides since the 1998 landslide.

Typhoon Morakot was one of the most severe typhoons to hit Taiwan. Nearly all inhabitants of steep mountainside communities in southern Taiwan were at risk of landslides. In total, the storm killed 650 people and caused US$3.3 billion worth of damage. A series of studies have used diagnostic interviews to examine the impacts of the disaster on 277 adolescents who were displaced as result of mudslides. Analyses of these interviews found that 25.8% of the adolescents had PTSD three months after the disaster. Female gender, being injured during the landslide, and bereavement as result of the disaster were all associated with increased risk of PTSD^30^.

The authors contrasted the prevalence of PTSD in their cohort with a similar school-based study carried out in Greece after an earthquake in 1999, when the prevalence of PTSD was 4.5% at the same time point (3 months) after the disaster. Some of the factors they consider may account for this difference include the characteristics of the subjects and differences in the methodologies used. The authors do consider that the type of disaster may account for some of the difference.

Notably, this research describes a small group of adolescents who had survived some of the worst impacts of the disaster. The participants were sent to boarding school as a means of providing shelter and a stable environment following destruction caused by the landslides. Their experiences are likely to be different from populations who do not have the opportunity to move away from the disaster zone or who are displaced to more difficult surroundings. Whilst they may have had better material surroundings, they may also have had reduced access to family support. Although the individuality of the experiences and the small sample size may limit generalisability, use of diagnostic interviews is an indicator of higher quality study and reliability of results.

The authors of two other papers about different aspects of the same study confirmed the reliability of the Multidimensional Anxiety Scale for Children in this setting^31^ and attempted to assess the direct and indirect effects of a variety of factors on suicide risk^29^. Female gender, more disaster-exposure experiences, and having PTSD and major depressive disorder were directly linked to greater suicide risk. Female gender and greater exposure to disasters were found to increase suicide risk indirectly. Subjects having perceptions of high levels of family support were found to have lower suicide risk^29^.

Difference of disaster type was also found to have an effect in longitudinal studies of survivors of a rainfall-induced disaster in Mexico in 1999 in which people from a locality that was affected primarily by landslides were compared with people from an area that had been affected by flooding. The survivors of the landslides had a higher prevalence of PTSD, as measured by diagnostic interview, at six months, which dropped quickly (from 46% at six months to 19% at 2 years post event). Survivors of floods had lower initial prevalence, but although the percentage reductions in their prevalence rates of PTSD over time were less (going from 14% at 6 months to 8% at 2 years), they were always lower than the rate for the landslides group. However, both rates were still at a level to prompt concerns about public mental health at the end of the two-year follow up period^28^. These findings are further complicated by both areas having higher levels of PTSD than the general population prior to the event, with the area affected by landslides having a higher PTSD prevalence following a previous event than the area affected by flooding.

In a second paper, the researchers reported that survivors of mass movements had a bigger deterioration in social support than those from the flood affected areas. Recovery of social support was slower among the groups of people that had survived landslides. Subjects who experienced landslides were more likely to have been bereaved (60.0% v 12.8%), lost larger amounts of property (58.5% v 44.2%), entered new conflicts (29.8% v 19.4%), or had changes in social networks (71.2% v 60.9%). They were less likely to have suffered injury or illness (23.9% v 60.9%) than those who were primarily exposed to floods. In general, women perceived that they had received less social support than did men^24^.

The role of family support in disasters was also studied after the landslides in Puerto Rico in 1985. Alcohol abuse, depression and total psychiatric symptomology were found to be higher when there was a lower level of emotional support. Although there was evidence that family roles were a predictor of effect, the authors’ opinion was that the relationship was more complex than they originally hypothesised.

The authors compared the Puerto Rican situation to that affecting survivors of a flood and resultant chemical release in St Louis, USA in the period from late 1982 through to early 1983. They report differences in response between the two groups, which may be due to their differing exposures. However, the differences might also have been due to differing cultural responses, differences in methodology when studying the two groups, as well as other unknown confounders^25^.

Culturally-specific responses to disasters are the subject of another study undertaken after the disaster in Puerto Rico in 1985. The study used modified diagnostic interviews to assess the prevalence of “*ataques de nervios*” (literally ‘attack of nerves’), described in the paper as a “Puerto Rican popular category of distress” characterised by symptoms including trembling, heart palpitations faintness and seizure-like episodes. Apparently, *ataques de nervios* are considered a normal expression of distress in Puerto Rico. Nonetheless, descriptions of the survivors’ symptoms appear substantial and to be sufficiently serious in some of the descriptions in the paper as to suggest that they would meet DSM diagnostic criteria. Exposure to mudslides was one factor that was associated with increased likelihood of *ataques de nervios*. Sufferers were much more likely to have significant mental disorders including PTSD, panic disorder or major depressive episodes. This study highlights the importance of awareness of cultural differences in how particular people respond when disasters occur^27^.

The psychosocial wellbeing and mental health of rescue workers and their families is also at risk during and after disasters. In a case report on providing support after landslides, Clifford (1999)^32^ describes the psychological support services provided after the Thredbo landslide in New South Wales, Australia, which resulted in 18 fatalities (including a member of the New South Wales Fire Brigade (NSWFB)). The labour-intensive and hazardous emergency response was complicated by a number of stressors including fatigue, frustration, fear for personal safety, personal knowledge of the victims, and media exposure. The NSWFB had a Crisis Incident Stress Management programme which relied on six interventions to provide support, needs assessment, on-scene crisis interventions, family interventions, group interventions, team debriefing and follow up, and longer term follow-up interventions. The feedback from professionals in relation to the work detailed in this case study indicates that rescue workers involved in this incident felt peer support was beneficial^32^.

That landslides have long-term consequences is shown in the 33-year follow up of the survivors of the Aberfan disaster, when a coal slag heap collapsed and engulfed part of a Welsh mining village, most notably including the local primary school. Survivors of the disaster were more likely to have had PTSD at some point since the disaster than controls (odds ratio 3.38, 95% confidence interval 1.40 – 8.47), and 29% (95% Ci 145-43) of survivors met the diagnostic criteria for PTSD at the 33 year follow up point^33^.

**Impact on healthcare**

Using our search strategy we identified only one study on the impact of landslides on healthcare provision. This is a case study of the impact of a series of landsides in Guatemala that not only buried a town, but also directly hit the small local hospital^34^. The case study describes the incident and the three phases of disaster response, starting, initially, with scattered, uncoordinated care; followed by centralized, coordinated care; and, later, arrival of outside aid and assistance. Perhaps surprisingly, the hospital was running again from an interim site within 16 days of the disaster. The study also details some of the factors that promoted successful movement from disaster response to recovery in this case, which included: starting the recovery process early with a shared vision; having key personnel who were invested in the survival of the hospital; ensuring local control of funds and existing pipelines for donations; and the presence of good links with external aid agencies.

## Discussion

The literature on landslides is rich with detailed technical geological and engineering papers covering spatial and temporal distributions of landslides; the processes through which they occur; their impacts in economic terms; and approaches to manage and mitigate their effects. Although it is well documented that deaths from landslides are substantial^3^^,^ 4, the published literature on the health impacts of these disasters is exceptionally limited. There are few studies that go beyond a simple statement of the number of fatalities, and even fewer that attempt to further describe or analyse the mortality or morbidity caused by landslides. We encountered this during the preperation of this review, with the main reason for rejecting a study being that it concentrated only on the technical aspects of landslides. This was also seen in the need to further limit the search in the SCOPUS dtabase as the volume of technical and engineering literature made searching over a longer timescale impractical.

Incorporation into a landslide exposes human bodies to a range of hazards, including blunt trauma, crushing, asphyxiation and drowning. Only one study has examined the causes of death in detail^16^. However, it is possible that the mechanisms of injury vary according to landslide type (for example, people incorporated into a wet landslide might be far more likely to suffer death through drowning than if they were involved in a dry flow) and rate of movement (with high rates perhaps being associated with a greater incidence of blunt trauma). However, these effects remain undocumented. We highlight one case report that describes in detail the mechanisms of death by reference to autopsy reports^35^, but it did not meet the eligibility criteria for this systematic review because the disaster took place in a deep mine, rather than it involving a surface landslide. This case is interesting because the force of the fast flowing mass movement was so great that it forced debris into all body cavities, causing suffocation and drowning as well as fatal trauma injuries. We conjecture that highly mobile flows on the surface may inflict similar injury patterns, but this is not documented.

The complexities of landslide motion are such that survival can occur when there appears to be little likelihood of escape. One example comes from the landslide in Hattian Bala Kashmir, Pakistan in 2005. Two women, who were cutting grass on the body of the rock avalanche prior to the event, survived the landslide unharmed: they remained on the surface of a large raft within the landslide mass as it moved downslope at velocities that exceeded 10 m/sec^36^. Zarroug *et al*. (2004) report that 30 cm of debris may impart sufficient force on the chest of a victim to overwhelm respiratory and diaphragmatic force if they are buried in beach sand^37^. Sanchez *et al*. have reported that, if protective structures are not sufficiently resilient, being inside them when they are incorporated into a landslide may increase rather than decrease the risks of people’s death^16^.

Attempts have been made to use mortality data to estimate risks from landslides, but there are limitations both in terms of the quality of the data and the ways in which they might be generalised due to differences between regions and populations. We think that caution should be taken when using risk estimates because of the concerns that have been raised about the validity of mortality estimates for some historical events, and the risks of event data not being captured.

The most researched aspect of landslide morbidity is the psychosocial and mental health impacts on survivors. There is a breadth of papers covering psychosocial matters, prevalence of psychiatric disorders and support for rescue staff. However, there is a preponderance of papers on people who develop PTSD. Further research would be valuable, especially on psychosocial resilience, the ways in which social support influences people’s mental health after landslides, and people who misuse substances and/or develop psychiatric disorders other than PTSD. Three of the papers, which relate to a single event, report studies on adolescents. Two other papers relate to a second event, such that there is a total of six events covered by the nine papers. It is important to differentiate between meeting the psychosocial needs of affected populations, which are most prominent in the immediate, short and medium term aftermath of disasters, and responding effectively to the medium and long term effects of disasters as risk factors for survivors and their relatives developing serious psychiatric disorders by providing timely and effective psychosocial care and mental healthcare. Psychosocial care relies predominantly on collective and public health orientated interventions whereas mental healthcare requires personalised care and treatment in additio 38. There is evidence that the people in need of mental healthcare who also receive psychosocial care have improved outcomes.

The ten papers on mental health and wellbeing we have reviewed report studies that used variable methodologies, which renders comparison between the papers difficult. Seven studies used diagnostic interviews, which produce greater diagnostic validity than do studies that are based on questionnaires alone.

Nonetheless, the papers suggest that survivors are more likely to develop serious mental disorders, such as PTSD, than control populations. Some comparisons also highlight that landslide survivors may have higher rates of PTSD than survivors of other disaster types. The evidence also demonstrates that there is a level of co-morbidity of PTSD and major depressive disorder, which is similar to findings in other disaster types. One possible explanation for these different patterns probably lies in the exceptionally energetic and violent nature of landslide events. It is also possible that this relates to a fear deep within humans of being buried alive^39^. Landslides are also less likely to have survivors than do other natural hazards, so there are fewer treatable injuries. Mass landslides lead to multiple bereavements and great destruction of property, which causes displacement, loss of social networks, loss of productive land, and other impacts that may worsen the immediate mental health impacts of any disaster. It is worth noting that the studies of Typhoon Morakot and the 1999 Mexico disaster suggest that there the loss of social support and the rates of PTSD may be associated.

The role of secondary stressors in psychosocial wellbeing post-disasters has been documented elsewhere^40^. Agencies and persons who respond to disasters should be aware of social as well as psychological and mental health impacts. Norris et al. (2005) discuss models of social support that suggest that social support after disasters is related to the initial severity and to the response in the immediate aftermath^24^. The community resilience model refers to the capacity of groups to overcome a shared trauma. There is an essential requirement to respond to disasters at a community level as well as an individual level.

Some of the papers on mental health were published some time ago, with only three of the nine papers published in the past five years. Two papers were published twenty years ago. Further advances in psychosocial care and disaster psychiatry have been made in the intervening period, making comparison of findings and generalisability more difficult. In particular, the diagnostic criteria for PTSD have changed several times since the earliest paper cited in this review, so some of the differences previously reported as ‘cultural’ may be considered differently if these studies were repeated today.

The report on support for rescue workers in New South Wales stresses the provision of a suite of measures to support emergency personnel and their families from the start of the crisis to long term follow up, which we believe is different to critical incident stress debriefing. However, the name is sufficiently similar for us to think it important to acknowledge recent research that has not shown evidence that single session individual psychological debriefing is beneficial, and may even increase rates of PTSD^41^. Nonetheless, psychosocial support for staff who intervene after disasters is very important. Recent advice on peer support for staff of rescue and other organisations is provided by a Delphi study from the Australian Centre for Posttraumatic Mental Health^42^^,^ 43.

The risk of an increase in infectious diseases is of concern during the relief and recovery phase after any major disaster. Displacement of people due to the destruction of their homes and other infrastructure can place them in unfamiliar surroundings, which, if they conflict with traditional beliefs and practices with regard to water supply and hygiene, can result in unsafe behaviours. The medium to long-term effects of changes to the environment caused by landslides, such as deforestation, and changes to river courses, can increase the risk of vector-borne diseases, and, as a result, the health impacts can extend long after the initial disaster has finished. Disruption of soil can also increase exposure to infectious organisms. It has been reported, for example, that an outbreak of coccidiomycosis occurred in the aftermath of large numbers of landslides triggered by an earthquake in 1994 in Southern California. The landslides, and the resultant bare landslide scars, caused soil particles to be released as airborne dust that contained the fungus Coccidioides immitis that was ingested by the population^44^.

The claim from one article included in the review that crush syndrome is the second most common cause of death in disasters is potentially problematic, given the difficulties in defining disasters and attributing cause of death. With the exception of crush syndrome, the lack of published information on the type and treatment of injuries caused by landslides means there is little evidence for rescuing and treating survivors that is directly related to disasters of the mass movement type. Improving the reporting of the health impacts of landslides and other mass movement disasters will greatly aid broader understanding of these events and help to improve government policies and relief and recovery efforts.

Overall, we believe that there is an urgent need to increase the number of studies of the mortality and nature of morbidity caused by landslides. The Micronesia study demonstrates that work of this nature is both viable and useful. Collation and analysis of existing autopsy reports as an initial step would be extremely valuable. Collating data in less developed countries, where the majority of landslide-related fatalities occur, is likely to be more challenging. However, we note that Micronesia is such a country, demonstrating that this kind of research is possible, even in this environment.

**Limitations**

The small number of papers that report studies that examined the health impacts makes it difficult to come to many firm conclusions about the health impacts of landslides. There may be a publication bias that results in only those landslides that cause many casualties, very large amounts of damage or receive significant media attention being seen as important enough to investigate or document the health impacts in detail.

Our decision to include only papers in the English language may have resulted in some key studies being excluded, particularly as literature may come from higher risk areas such as Turkey, South America, and China and other countries in the Himalayan region.

Other limitations of the search strategy include the SCOPUS search being limited to articles published in the past 20 years, and removal of the search string that was intended to identify damage to key healthcare infrastructure.

**What was already known**

There is ample evidence in the literature that landslides are natural hazards that occur widely around the world. Recent studies of mortality due to landslides have highlighted human deaths as being substantially higher than had been previously estimated, and that there are a number of key hotspots for landslide impacts^4^. Mostly, they occur in poor countries with steep terrain, such as the southern edge of the Himalayan arc. Increasingly, the science of landslide physics is allowing the nature of these hazards to be understood, which is leading to better techniques through which they can be managed and mitigated. In many cases, this has had a startling impact. In Hong Kong, for example, fatalities have dropped from about 50 per year in the mid-1970s to one or two per decade now^3^.

It is also established that landslides can have a range of health impacts in addition to causing fatalities.

**What this study adds**

This study systematically reviews, for the first time, the literature on the health impacts of landslides. It demonstrates that, although it is scientifically viable to investigate these effects, there is a surprising paucity of studies that have done so. Thus, it is clear that our understanding of the causes of death, the nature of injuries, and the long-term health impacts of landslides remains at a very low level. Given that landslides are likely to increase in frequency in the future, it is crucial that further studies are undertaken of the health impacts of landslides.

## Conclusions and recommendations

We conclude that the health impacts of landslides are poorly documented in almost all respects. Causes of death and the nature of injuries suffered in landslides remain almost entirely undocumented in the literature, and there are very few studies of the nature of treatments required by the victims of landslides.

A greater level of information is available with respect to the psychiatric impacts. These studies show that the effects are more substantial than for other types of natural hazards. Probably, this reflects the violence of landslides, but even this evidence is limited. Consequently, we recommend that further studies of this type are undertaken, spanning a range of landslide types and a range of human and physical environments. These studies should inform policy for healthcare responses and services; the built environment; and transport. This research is also required if people who respond to landslides are to be equipped with the knowledge and tools they require to maximise survival rates.

## Appendix 1

### PRISMA checklist

**Table:** Page numbers refer to original manuscript

**Section/topic **	**#**	**Checklist item **	**Reported on page # **
**TITLE **			
Title	1	Identify the report as a systematic review, meta-analysis, or both.	1
**ABSTRACT **			
Structured summary	2	Provide a structured summary including, as applicable: background; objectives; data sources; study eligibility criteria, participants, and interventions; study appraisal and synthesis methods; results; limitations; conclusions and implications of key findings; systematic review registration number.	1
**INTRODUCTION **			
Rationale	3	Describe the rationale for the review in the context of what is already known.	2-3
Objectives	4	Provide an explicit statement of questions being addressed with reference to participants, interventions, comparisons, outcomes, and study design (PICOS).	3
**METHODS **			
Protocol and registration	5	Indicate if a review protocol exists, if and where it can be accessed (e.g., Web address), and, if available, provide registration information including registration number.	Protocol available from authors
Eligibility criteria	6	Specify study characteristics (e.g., PICOS, length of follow-up) and report characteristics (e.g., years considered, language, publication status) used as criteria for eligibility, giving rationale.	3-4
Information sources	7	Describe all information sources (e.g., databases with dates of coverage, contact with study authors to identify additional studies) in the search and date last searched.	3
Search	8	Present full electronic search strategy for at least one database, including any limits used, such that it could be repeated.	23
Study selection	9	State the process for selecting studies (i.e., screening, eligibility, included in systematic review, and, if applicable, included in the meta-analysis).	4
Data collection process	10	Describe method of data extraction from reports (e.g., piloted forms, independently, in duplicate) and any processes for obtaining and confirming data from investigators.	4
Data items	11	List and define all variables for which data were sought (e.g., PICOS, funding sources) and any assumptions and simplifications made.	4
Risk of bias in individual studies	12	Describe methods used for assessing risk of bias of individual studies (including specification of whether this was done at the study or outcome level), and how this information is to be used in any data synthesis.	N/A
Summary measures	13	State the principal summary measures (e.g., risk ratio, difference in means).	N/A
Synthesis of results	14	Describe the methods of handling data and combining results of studies, if done, including measures of consistency (e.g., I^2^) for each meta-analysis.	N/A

**Table:**

**Section/topic **	**#**	**Checklist item **	**Reported on page # **
Risk of bias across studies	15	Specify any assessment of risk of bias that may affect the cumulative evidence (e.g., publication bias, selective reporting within studies).	N/A
Additional analyses	16	Describe methods of additional analyses (e.g., sensitivity or subgroup analyses, meta-regression), if done, indicating which were pre-specified.	N/A
**RESULTS **			
Study selection	17	Give numbers of studies screened, assessed for eligibility, and included in the review, with reasons for exclusions at each stage, ideally with a flow diagram.	22
Study characteristics	18	For each study, present characteristics for which data were extracted (e.g., study size, PICOS, follow-up period) and provide the citations.	19-21
Risk of bias within studies	19	Present data on risk of bias of each study and, if available, any outcome level assessment (see item 12).	N/A
Results of individual studies	20	For all outcomes considered (benefits or harms), present, for each study: (a) simple summary data for each intervention group (b) effect estimates and confidence intervals, ideally with a forest plot.	N/A
Synthesis of results	21	Present results of each meta-analysis done, including confidence intervals and measures of consistency.	N/A
Risk of bias across studies	22	Present results of any assessment of risk of bias across studies (see Item 15).	N/A
Additional analysis	23	Give results of additional analyses, if done (e.g., sensitivity or subgroup analyses, meta-regression [see Item 16]).	N/A
**DISCUSSION **			
Summary of evidence	24	Summarize the main findings including the strength of evidence for each main outcome; consider their relevance to key groups (e.g., healthcare providers, users, and policy makers).	10-12
Limitations	25	Discuss limitations at study and outcome level (e.g., risk of bias), and at review-level (e.g., incomplete retrieval of identified research, reporting bias).	13
Conclusions	26	Provide a general interpretation of the results in the context of other evidence, and implications for future research.	13-14
**FUNDING **			
Funding	27	Describe sources of funding for the systematic review and other support (e.g., supply of data); role of funders for the systematic review.	14

*From: * Moher D, Liberati A, Tetzlaff J, Altman DG, The PRISMA Group (2009). Preferred Reporting Items for Systematic Reviews and Meta-Analyses: The PRISMA Statement. PLoS Med 6(6): e1000097. doi:10.1371/journal.pmed1000097 
